# Logic of Selecting Suitable Dissolution Parameters in New Drug Formulations Based on A BCS Approach

**DOI:** 10.22037/ijpr.2020.1100993

**Published:** 2020

**Authors:** Raúl Medina-López, Edgar E. Arregui, Edson J. Aranda, Luis A. Moreno-Rocha, Marcela Hurtado, Helgi Jung-Cook, Sally A. Helmy

**Affiliations:** a *Departamento de Sistemas Biológicos, Universidad Autónoma Metropolitana-Xochimilco, Mexico City, Mexico. *; b *Departamento de Farmacia, Facultad de Química, Universidad Nacional Autónoma de México, Mexico City, Mexico. *; c *Department of Clinical and Hospital Pharmacy, Faculty of Pharmacy, Taibah University, AL-Madinah AL-Munawarah, Kingdom of Saudi Arabia. *; d *Department of Pharmaceutics, Faculty of Pharmacy, Damanhour University, Damanhour, Egypt.*

**Keywords:** Carbamazepine, Dose, Metronidazole, Propranolol-HCl, Ranitidine-HCl, Reference products

## Abstract

Since the biopharmaceutical quality of generic drug formulations depends on the quality of the reference products and also information about the *in-vitro* release performance of drugs under different conditions is scarce in the literature, a dissolution study of four reference tablets was performed. Each drug was representative of one Class of the Biopharmaceutical Classification System. The *in-vitro* release performance of propranolol-HCl, carbamazepine, ranitidine-HCl, and metronidazole was evaluated using a USP basket and paddle apparatus at different agitation rates (50, 75, and 100 rpm) with two doses of each drug. In all experiments, pharmacopeial dissolution media was used and the samples were taken with automatic equipment at specific times up to 60 min, except for propranolol-HCl, for which the samples were taken up to 30 min. The dissolution profiles were compared by model-independent, model-dependent, and ANOVA-based comparisons. The three methods of data comparison showed that low *vs*. high doses were significantly different (*P <* 0.05), which may influence cases in which biowaivers of propranolol-HCl and ranitidine-HCl are requested. Additionally, the results showed that despite different hydrodynamic environments produced by the basket and paddle apparatus, under certain conditions, both types of equipment generated comparable *in-vitro* results. Variables such as the dose, agitation rate, and type of dissolution apparatus are important factors to consider in designing dissolution tests for drug products. This information can be used to test a new dosage when there is no pharmacopeial method available to perform a dissolution study. Further researches on the *in-vitro* release performance of reference drug products are required.

## Introduction

Dissolution studies are used to describe the *in-vitro* release performance of drug products. The appropriate choice of dissolution equipment, agitation rate, and dissolution media is essential to designing a method that is able to reflect quality differences among pharmaceutical products. The common equipment used to carry out a dissolution study is USP Apparatus 1 (basket) or USP Apparatus 2 (paddle). Of the dissolution methods included in the FDA Dissolution Methods Database, 17% use the basket apparatus and 70% use the paddle apparatus (1). In this database, the agitation rate for the basket apparatus ranges from 35 to 200 rpm, while that of the paddle apparatus ranges from 25 to 200 rpm. To support the selection of a dissolution test, the influence of the hydrodynamic flow generated by different agitation rates and USP apparatus, among other conditions, has been evaluated by some authors (2‒6).

On the other hand, generic drug products are commonly used because of their low cost and wide availability. The good quality of these products is important for patients, health centers, and the pharmaceutical industry. To ensure the quality of generic drugs, these products must be compared with reference products whose safety and efficacy has been demonstrated. A complete evaluation of the new drug products includes *in-vitro* dissolution and *in-vivo* studies. The comparison of the *in-vitro* release behavior between generic and reference products can be good or bad depending on how much information is available from references. *In-vivo* studies are the best way to establish the adequate performance of generic drugs, but due to the high cost of bioequivalence studies and the impact of the Biopharmaceutics Classification System (BCS) approach in drug development, the FDA Guidelines for Industry (based on BCS) indicate the criteria by which bioequivalence studies can be replaced by *in-vitro* dissolution studies (7).

Propranolol-HCl, carbamazepine, ranitidine-HCl, and metronidazole are widely used as generic drugs and are included in the WHO model list of essential medicines (8). According to the BCS, propranolol-HCl and carbamazepine are Class I (owing to the high solubility/high permeability of propranolol-HCl) and Class II (owing to the low solubility/high permeability of carbamazepine) drugs, respectively (9). Ranitidine-HCl is a Class III drug (owing to its high solubility/low permeability) (10). Some authors have classified metronidazole as a Class I drug (9, 11), and others have classified it as a Class III drug (12). Conversely, TSRL database reports metronidazole as a Class IV drug (low solubility/low permeability) (13, 14). Biowaiver monographs for propranolol-HCl (15), ranitidine-HCl (10), and metronidazole tablets (11) have been previously published. To date, no biowaiver monograph for carbamazepine tablets is available. Additionally, information about the effect of USP dissolution apparatus and/or the agitation rate on different doses of reference tablets of propranolol-HCl, carbamazepine, ranitidine-HCl, and metronidazole is scarce (16, 17).

Descriptions of the *in-vitro* release of the drugs have been carried out by mathematical models (18‒20). The purpose of using mathematical models is that they facilitate the analysis and interpretation of the observed data because they describe the dissolution profiles as a function of only a few model parameters that can be statistically compared (21). One of the most commonly cited mathematical models is the Weibull function since this equation can be successfully applied to almost all types of dissolution curves and is commonly used to describe the *in-vitro* release of some drugs (18, 22). Dissolution profiles are often compared by analysis of variance (ANOVA)-based, model-dependent, and model-independent methods (23‒25). Each comparison has its advantages by providing different information on the rate and extent of *in-vitro* drug dissolution.

The objective of this study was to evaluate the influence of the dose, agitation rate, and dissolution apparatus on the *in-vitro* release performance of reference products containing propranolol-HCl, carbamazepine, ranitidine-HCl, and metronidazole. The USP basket, paddle apparatus, and two doses of each drug product were tested at 50, 75, and 100 rpm. The dissolution profiles of all of the drugs were compared by the three methods mentioned above. This information can help to establish experimental conditions that allow the determination of differences in the quality of generic drugs or can be used to test a new dosage when there is no pharmacopeial method available to perform a dissolution study.

## Experimental

*Drug products and reagents*

Two doses of propranolol-HCl (Inderalici 10 and 40 mg), carbamazepine (Tegretol 200 and 400 mg), ranitidine-HCl (Azantac 150 and 300 mg), and metronidazole tablets (Flagyl 250 and 500 mg) were used. These products were selected as reference products for bioequivalence studies by the Mexican regulatory agency (26). Hydrochloric acid and ethanol were analytical grade (J.T.Baker®, Mexico), and sodium lauryl sulfate (Distribuidora Quimica Lufra, Mexico) was purchased from local suppliers. Reference standards of the four drugs were purchased from Sigma-Aldrich Co. (St. Louis MO, USA). All dissolution samples were filtered through 0.45 µm nitrocellulose filters (Millipore®, Ireland).

*Content uniformity and assay*

Content uniformity and assay tests were performed according to the procedures described in the United States Pharmacopeia (27).

*In-vitro release studies*

Dissolution data for propranolol-HCl and ranitidine-HCl tablets were obtained with an automated USP Apparatus 1 and 2 (Sotax AT7-Smart, Switzerland) coupled to a piston pump (Sotax CY-7, Switzerland) and a UV/Vis spectrophotometer (Perkin Elmer Lambda 25, USA). Dissolution profiles of carbamazepine and metronidazole tablets were obtained with an automated USP Apparatus 1 and 2 (Vankel VK 7000, Erweka, Germany) coupled to a multi-channel peristaltic pump (Vankel VK 810, England) and a UV/Vis spectrophotometer (Varian Cary 50 Tablet, USA). Table 1 lists the current pharmacopeial conditions for the different drug products used (27, 28). In this work, the same dissolution media were used, but at different agitation rates (50, 75, and 100 rpm). The samples were automatically withdrawn every two minutes up to 60 min, except for propranolol-HCl, for which the samples were taken up to 30 min. The dissolved amount of each drug was determined by comparing the absorbances of the samples with their respective standard solutions.

*Data analysis*

Dissolution profiles were compared by ANOVA-based, model-dependent, and model-independent methods (23‒25). For model-independent comparisons, the quantity of the drug dissolved at a specific time (Q) mean dissolution time (MDT) as well as the dissolution efficiency (DE) were calculated and compared with Student’s *t*-tests. Differences were considered significant if *P <* 0.05. For model-dependent comparisons, all dissolution data were fitted to the single rectangular hyperbola model (Equation 1) and Weibull function (Equation 2). The single rectangular hyperbola model has been used to describe the *in-vitro* release of carbamazepine tablets and benzoyl metronidazole suspensions (29). The time required to dissolve 63.2% of the dose (t_63.2%_) derived from data fitted to the rectangular hyperbola model and time parameter (Td) derived from data fitted to the Weibull function was also determined. Td is defined as the time interval necessary to dissolve 63.2% of the drug (30). Both model-dependent parameters were used for statistical comparisons in the same way as the model-independent comparisons were performed. The MDT and DE values as well as Weibull fitting were calculated using the Excel add-in DDSolver program (31), and the rectangular hyperbola parameters were calculated by SigmaPlot software (version 11.0).

$y=\frac{\mathrm{ax}}{b+x}$                      (Equation 1)

$F=100\left[1-e^{-\frac{{\left(t-\mathrm{Ti}\right)}^{\beta }}{\alpha }}\right]$                      (Equation 2)

where y is the percentage of drug released at time x and a and b are constants for Equation 1. For Equation 2, F is the percentage of drug released at time t; α is the scale parameter, which defines the time scale of the process; β is the shape parameter, which characterizes the curve; and Ti is the location parameter, which represents the lag time before the onset of the dissolution process and, in most cases, will be near zero (31).

ANOVA-based comparisons were planned with a general full factorial design. Three independent variables were stipulated: dose (mg), agitation rate (rpm), and dissolution apparatus (basket or paddle). The tests were fully randomized to eliminate any bias in the results. For these comparisons, only DE values were considered, and they were taken as dependent variables. The effects of interactions between the factors were included in a model in which 12 possible combinations were evaluated. Finally, the influence of variables was analysed by means of statistical calculations, including three-way ANOVA followed by a Holm-Sidak multiple comparison test. All statistical calculations were carried out using SigmaStat software (version 3.5). Differences were considered significant if *P <* 0.05.

## Results and Discussion

*Content uniformity and assay*

All drug products underwent the quality control tests described in the USP. The results are shown in Table 2.

*Model-independent comparisons*

**Figure 1:**
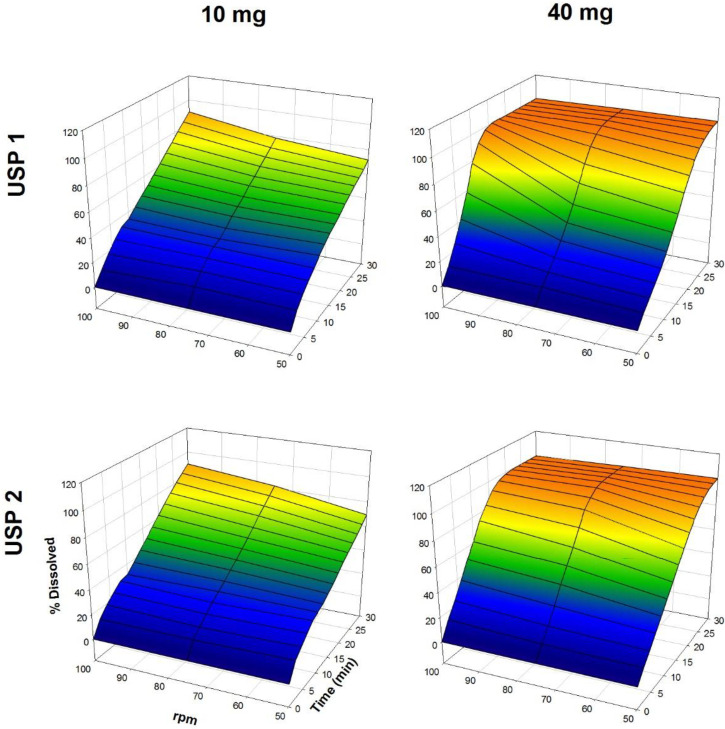
Dissolution profiles of propranolol-HCl tablets. Each point is the average of six determinations

**Figure 2:**
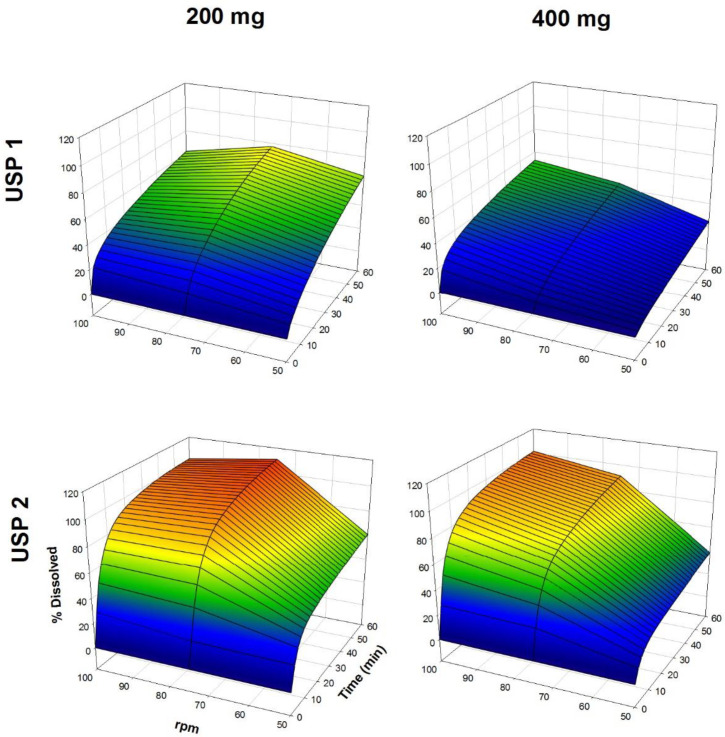
Dissolution profiles of carbamazepine tablets. Each point is the average of six determinations

**Figure 3:**
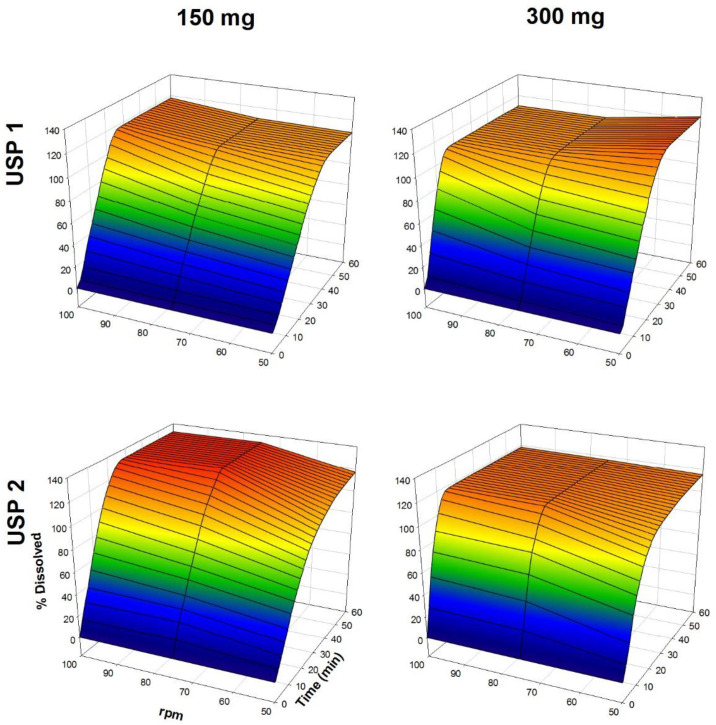
Dissolution profiles of ranitidine-HCl tablets. Each point is the average of six determinations

**Figure 4:**
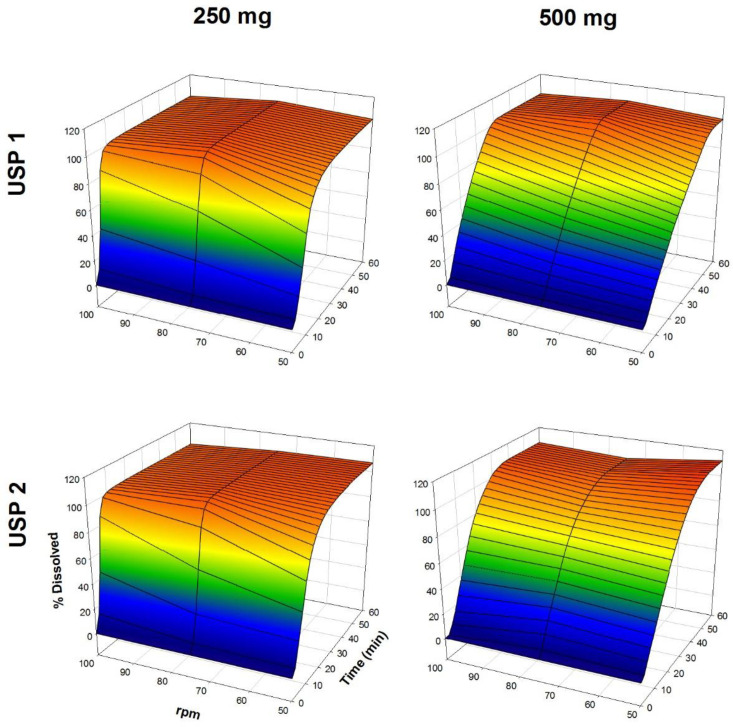
Dissolution profiles of metronidazole tablets. Each point is the average of six determinations

The dissolution profiles of propranolol-HCl, carbamazepine, ranitidine-HCl, and metronidazole are shown in Figures 1-4, respectively. Data are shown on a three-dimensional plot in which the dissolved percentage of each drug, as a function of time and agitation rate, is easily observed.

Some authors found that a three-dimensional plotting technique used to characterize the *in-vitro* release performance was an adequate tool for delineating the properties of dissolution rate of the drugs with bioavailability problems (2, 4). Others, with the aim of finding better dissolution tests, tested USP Apparatus 1 and 2 at 50 and 100 rpm (32, 33).

**Figure 5:**
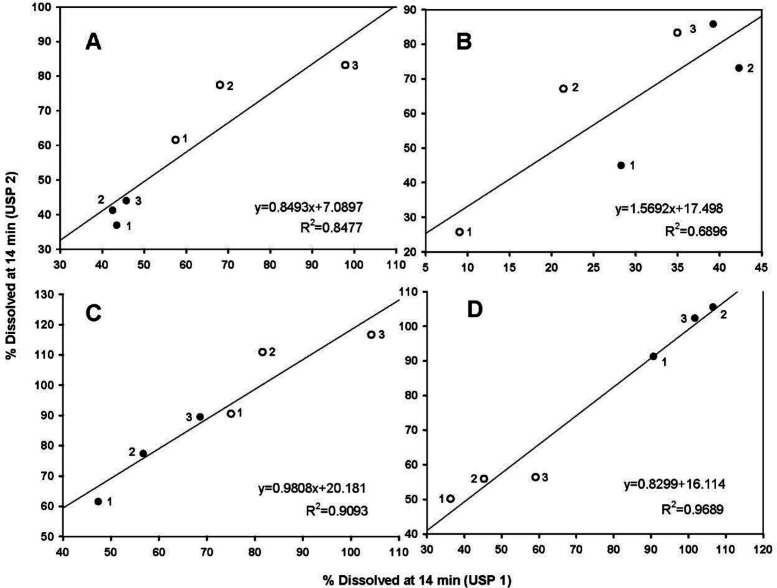
Comparison of dissolution rates for all drug products. (A) propranolol-HCl, (B) carbamazepine, (C) ranitidine-HCl, and (D) metronidazole tablets. (●) low dose, (○) high dose, (1) 50 rpm, (2) 75 rpm, and (3) 100 rpm. Each point is the average of six determinations

Because the hydrodynamic flow characteristics may be useful for understanding differences or similarities of the dissolution behavior of pharmaceutical products (4, 5), the dissolved percentages at 14 min between the basket and paddle apparatus were taken, as being indicative of the dissolution rate, and plotted. Figure 5 shows the data.

All data were fitted to a linear mathematical model (y = mx + b). Morihara *et al*. (5), used the same technique. As shown, metronidazole data were better adjusted to a straight-line equation with the highest determination coefficient (R^2^ = 0.9689), whereas carbamazepine data had the lowest determination coefficiente (R^2^ = 0.6896). The highest determination coefficient of metronidazole suggests that the tablets have linear dissolution characteristics between both USP apparatuses (Figure 5D).

Very rapid drug dissolution can be achieved if ≥ 85% of the drug is dissolved in 15 min or less, but rapid drug dissolution can be achieved if the same percentage of drug dissolution is reached in 30 min or less (34, 35). In the case of USP Apparatus 1, propranolol-HCl, ranitidine-HCl, and metronidazole were dissolved over a range of 40-100% at 14 min (Figure 5B), whereas by that time, only 10-45% of carbamazepine was dissolved. The aqueous solubility of carbamazepine is 237.2 ± 5.2 µg/mL at 37 °C, and a 10-fold increase in solubility has been reported with sodium lauryl sulfate aqueous solutions (36). The solubility of metronidazole is 64.8 mg/mL at pH 1.2 (11). Due to the solubility of carbamazepine, dissolution media, and agitation rate of 50 to 100 rpm, an increase in the dissolved percentage at 14 min was expected, but the opposite was observed.

The Q, MDT, and DE average ± standard error medium (SEM) values are shown in Table 2. Propranolol-HCl, carbamazepine, and metronidazole tablets met the pharmacopeial criterion (Q). To evaluate the influence of dose, dissolution profiles obtained under the same agitation rate and type of apparatus conditions were compared (low *vs*. high dose). Additionally, to determine the influence of the type of apparatus, the dissolution profiles obtained under the same conditions of the dose and agitation rate were also compared (USP 1 *vs*. USP 2).

A dose effect was observed for all of the drug products. Comparing the three model-independent parameters of the four drugs, low and high dose were significantly different (*P <* 0.05). The Q and MDT values were similar for both propranolol-HCl doses and the low dose of metronidazole (*P* > 0.05). Conversely, for the rest of the results, significant differences were observed in at least one comparison (*P <* 0.05). The investigated drugs were ranked with respect to the significant differences in the recorded values of the three model-independent parameters as propranolol-HCl < metronidazole < ranitidine-HCl < carbamazepine. This finding was consistent with the solubility of propranolol-HCl (1 g in 10-30 mL of water) (15) and carbamazepine, where it was expected that propranolol-HCl might be the drug with the smallest number of differences (39% of a total of 18 comparisons), while carbamazepine has the largest number of differences (94% of a total of 18 comparisons).

The few differences observed for propranolol-HCl tablets, as well as its classification as a Class I drug, should not be overestimated in manufacturing a generic drug product. An *in-vitro* dissolution study of propranolol-HCl tablets (USP Apparatus 2, 50 rpm) with two generic products and reference (40 mg) showed that even though all of the drug products dissolved ≥ 85% within 30 min, none of the tested products had f_2_ ≥ 50 in the three-dissolution media used (900 mL of pH 1.2, 4.5, and 6.8) (37). On the other hand, for ranitidine-HCl, 89% differences were found (solubility of 660 mg/mL in water at room temperature) (10). For metronidazole, 50% differences were observed despite being a low solubility/low permeability drug. The delay of the dissolution performance of carbamazepine, ranitidine-HCl, and metronidazole tablets could be attributed to excipients or manufacturing process.

Comparing the dissolution profiles using a model-independent approach, the dissolution of low *vs*. high doses of the four drug products studied was significantly different. On the other hand, out of a total of 24 comparisons of each model-independent parameter, 37% of Q data and 30% of MDT and DE data supported the idea that despite the different hydrodynamic environments of basket and paddle apparatus, at a specific agitation rate and/or under specific dose conditions, both types of equipment generated comparable *in-vitro* results. The data used for these comparisons are important because the Q criterion is a quality parameter that is widely used in pharmaceutical manufacturing. MDT is defined as the time to reach 63.2% of drug dissolution or the average value of a log-normal distribution (4), and some authors use DE as a suitable parameter that express the global drug dissolution performance, which is useful for comparing dissolution profiles (38). In addition, the MDT and DE values are considered to be appropriate parameters for *in-vitro/in-vivo* correlation for level B and level C, respectively (39).

*Model-dependent comparisons*

Hyperbola (a and b) and Weibull (α and β) parameters as well as the t_63.2%_ and Td values are shown in Table 3. The Td value is calculated with α and β parameters and is equivalent to MDT (40). Considering an adjusted determination coefficient (R^2^_adj_) > 0.99, 30% of data were better adjusted to a single rectangular hyperbola model and 63% were better adjusted to a Weibull function.

Comparing low and high doses according to the t_63.2% _and Td values, significant differences were found under all conditions used for propranolol-HCl, ranitidine-HCl, and metronidazole tablets and for almost all Td data of carbamazepine (*P <* 0.05), whereas the t_63.2%_ values of carbamazepine were similar (*P* > 0.05). Concerning the type of apparatus, the drugs were ranked with respect to significant differences in the t_63.2% _and Td values as metronidazole < propranolol-HCl < ranitidine-HCl. No significant differences were found (*P* > 0.05) with the model-dependent parameters of low dose of metronidazole, while all ranitidine-HCl comparisons were significantly different (*P <* 0.05). For both doses of propranolol-HCl and the high dose of metronidazole, significant differences were observed in at least one comparison (*P <* 0.05). Due to the high solubility of propranolol-HCl and ranitidine-HCl, it was expected that both drugs would be less different than metronidazole, but the opposite was observed. In all drugs, R^2^_adj_ values calculated with the single rectangular hyperbola equation and the Weibull function ranged between 0.8596-0.9988 and 0.9079-0.9995 except for the R^2^_adj_ values for ranitidine-HCl calculated by the Weibull function, which ranged from 0.3868 to 0.9677 (Table 3).

Out of a total of 18 comparisons of each model-dependent parameter, 40% of data supported the idea that the basket and paddle apparatus generated comparable *in-vitro* results. For this assertion, no data from carbamazepine were considered. Most of the t_63.2%_ and Td values of carbamazepine were unrealistic, with negative signs or values that were too high (*e.g.*, t_63.2%_ of 140 h for 400 mg, 75 rpm, and the basket apparatus) despite similar and good fit to both equations (R^2^_adj_ 0.9666-0.9988 for the rectangular hyperbola equation and 0.9749-0.9995 for the Weibull function) (Table 3). This issue could be attributed not only to the hydrodynamic environment generated by both USP apparatuses but also to the low solubility of carbamazepine, type and amount of excipients used or the manufacturing process itself. If the results of a derived parameter of any fitting are illogical, comparison of dissolution profiles by the model-dependent approach is not recommended. Several problems have been reported with the official carbamazepine dissolution test. Jung *et al*. (41) found that no *in-vitro/in-vivo* correlation, using pharmacopeial conditions, was obtained for carbamazepine tablets Moreover, Medina *et al*. (42) reported that the flow-through cell method (USP Apparatus 4) allowed to differentiate better between generic drug products than the official dissolution test.

**Figure 6:**
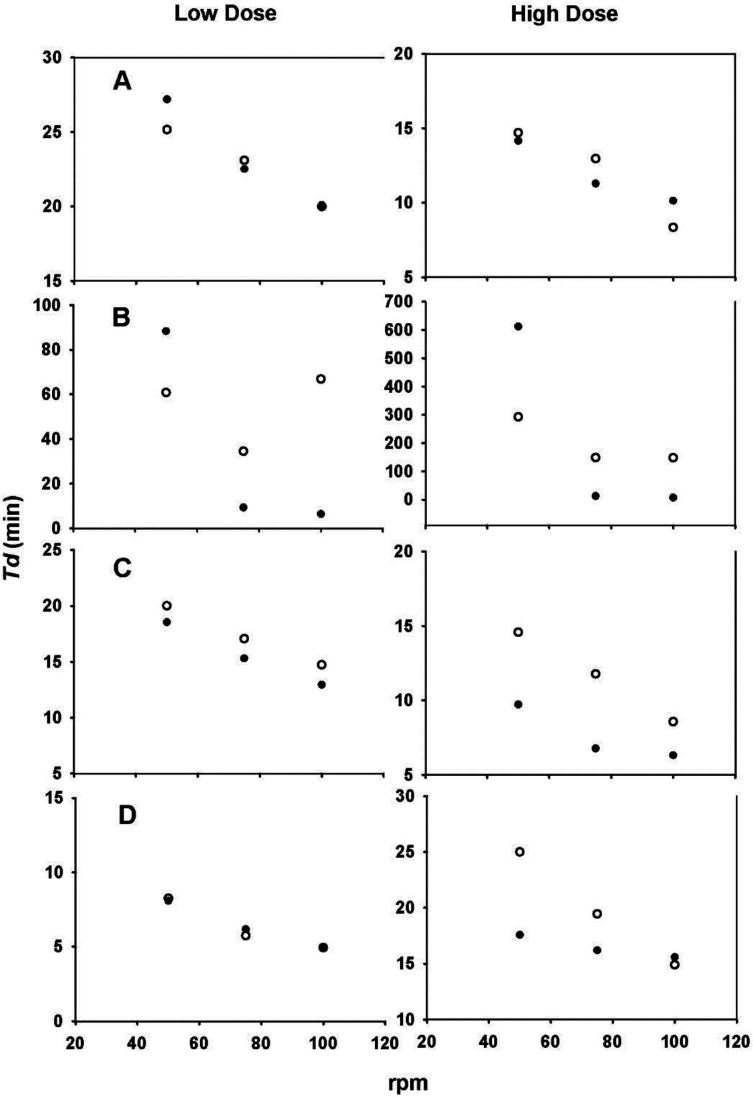
Td values *vs*. agitation rate. (A) propranolol-HCl, (B) carbamazepine, (C) ranitidine-HCl, (D) metronidazole tablets, (●) paddle and (○) basket. Each point is the average of six determinations

With the aim of evaluating the hydrodynamic dependence of each drug, Td values as a function of the agitation rate were plotted. Scholz *et al*. used the same technique (6). The results are shown in Figure 6. The changes in Td values observed in all plots clearly show the hydrodynamic dependence of both doses of the four drugs. The greatest differences were found with the carbamazepine data (Figure 6B).

The Td values were inversely proportional to the agitation rate, where a decrease in Td values was observed because of an increase in the agitation rate, in some cases resulting in an apparent mathematical relationship. An advantage of using the Weibull function is that the parameter calculation is independent of whether sink conditions prevail. Moreover, changes in Td indicate a dependence of the dissolution process on the hydrodynamics of the system used (6).

The single rectangular hyperbola model can mathematically describe the common shape of a dissolution profile. The t_x%_ value, such as t_80%_ (represents time to achieve 80% drug dissolution), is easily calculated with a and b parameters derived from the data adjusted to this model. Additional information can be obtained with the Weibull function. The shape parameter (β) characterizes the dissolution profile as exponential (β = 1), sigmoid (S-shaped), with an upward curvature followed by a turning point (β > 1), or parabolic, with a steeper initial slope consistent with the exponential (β < 1) (16). In our work, dissolution data from all of the investigated drugs were fitted to a hyperbola model and Weibull function, and the latter model showed that the dissolution profiles of all of the drugs were sigmoid, except those of carbamazepine, for which the parabolic shape prevailed because of β values < 1. The results were consistent with those previously reported where carbamazepine reference and some generic drug products (200-mg tablets) had β values < 1 using USP Apparatus 2 (75 rpm) and USP Apparatus 4 (laminar flow at 16 mL/min) (42).

Comparisons of the low *vs*. high dose of the four drug products studied showed significant differences in their *in-vitro* release performance. An unexpected situation was found with Class I and Class III drugs, and this finding could dispute the biowaivers for low doses of propranolol-HCl and ranitidine-HCl. Additionally, almost 50% of the data derived from the two adjustments supported the idea that under certain agitation rates and/or doses, both USP apparatuses produced equivalent *in-vitro* results.

*ANOVA-based comparisons*

ANOVA-based comparisons were also used to compare the dissolution profiles of propranolol-HCl, carbamazepine, ranitidine-HCl, and metronidazole tablets. The advantage of this approach is that it is not restricted to any of the requirements of model-independent comparisons, and in addition, it does not depend on data fitting to a specific equation. Comparisons were carried out with the DE values taken as the extent of the dissolution behavior. For each drug product, the *P*-values from the three-way ANOVA are shown in Table 4.

As observed in Table 4, due to the use of different dissolution apparatus (as a main factor), significant differences were observed for carbamazepine and metronidazole tablets (*P <* 0.001). Significant differences in all drug products, as a result of the use of different agitation rates and doses (as main factors), were found (*P <* 0.001). Considering an increase in the agitation rate (50-100 rpm) and different hydrodynamic patterns generated by the basket and paddle apparatus, significant differences with these factors were expected. However, the influence of the dose seems to be of more importance, especially if a biowaiver of a low dose tablet is required.

Significant interactions between factors, such as the dissolution apparatus, agitation rate, and agitation rate and dose, were found for all drug products (*P <* 0.05). A significant interaction between the dissolution apparatus and dose was observed only for carbamazepine tablets (*P <* 0.05). In all of the drug products, except metronidazole tablets, a significant interaction with the three factors studied was observed (*P <* 0.001). It is important to consider significant differences in dose as a factor. As a result, propranolol and ranitidine tablets manufactured as salts could improve the dissolution of these drugs. The volume and type of dissolution media used in dissolution tests of these tablets should adequately dissolve both doses, but the quality of excipients and the manufacturing process are critical factors that control drug release.

According to Table 4, significant differences in all sources were found for carbamazepine (*P <* 0.05). Accordingly, special attention should be paid to the design of a dissolution test for carbamazepine tablets because carbamazepine has high inter-variability and a narrow therapeutic window. Therefore, it is not a candidate for a biowaiver. In the case of metronidazole, significant differences in all sources were observed (*P <* 0.001), except for the interaction of the dissolution apparatus and dose and interactions of the three factors involved (*P* > 0.05). Metronidazole’s basic nature and the acidity of the dissolution medium were not enough to adequately dissolve both doses used. The DE range for the metronidazole low dose was 88.2-97.6%, while for the high dose, it was 65.50-81.20%. Again, the manufacturing process plays an important role in the *in-vitro* release of metronidazole tablets. These results were consistent with those reported by Medina *et al*. (42), where metronidazole reference tablets and some generic drug products (250-mg and 500-mg) had different release patterns using the USP basket apparatus (100 rpm) and flow-through cell method (laminar flow at 24 mL/min). As was indicated previously, information regarding metronidazole classification is controversial, and the results obtained in this work on model-independent, model-dependent, and ANOVA-based comparisons support this controversy.

**Figure 7:**
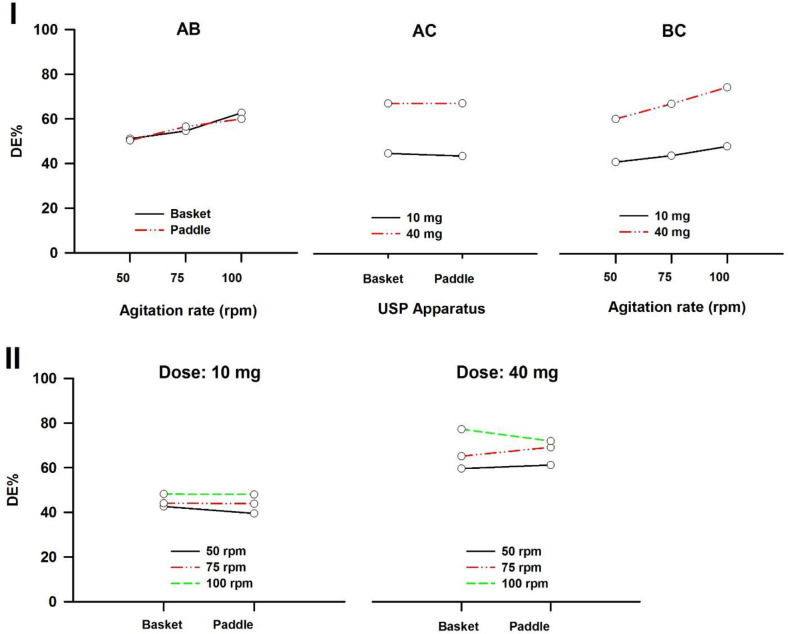
Interaction plots of propranolol-HCl tablets. (I) First-order and (II) Second-order. A: Dissolution apparatus, B: agitation rate, and C: dose

**Figure 8:**
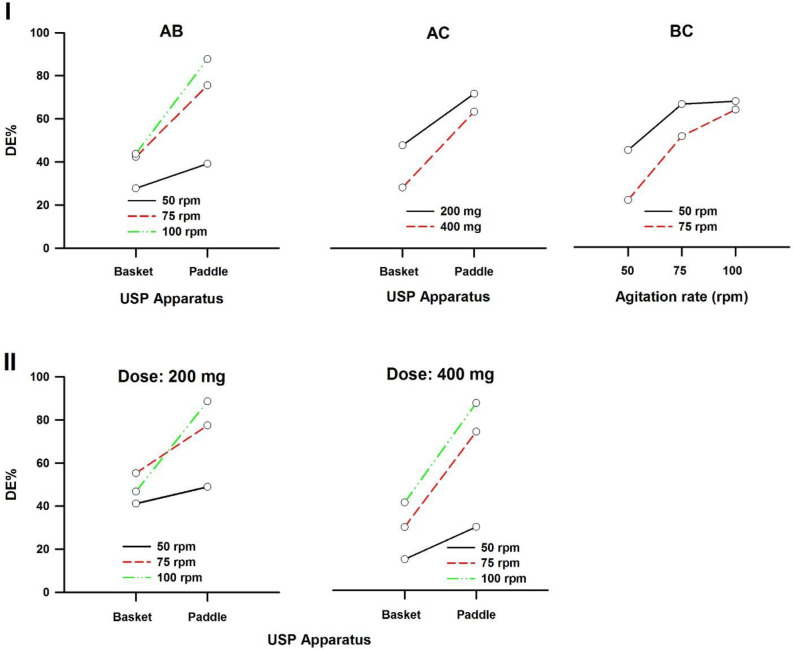
Interaction plots of carbamazepine tablets. (I) First-order and (II) Second-order. A: Dissolution apparatus, B: agitation rate, and C: dose

**Figure 9:**
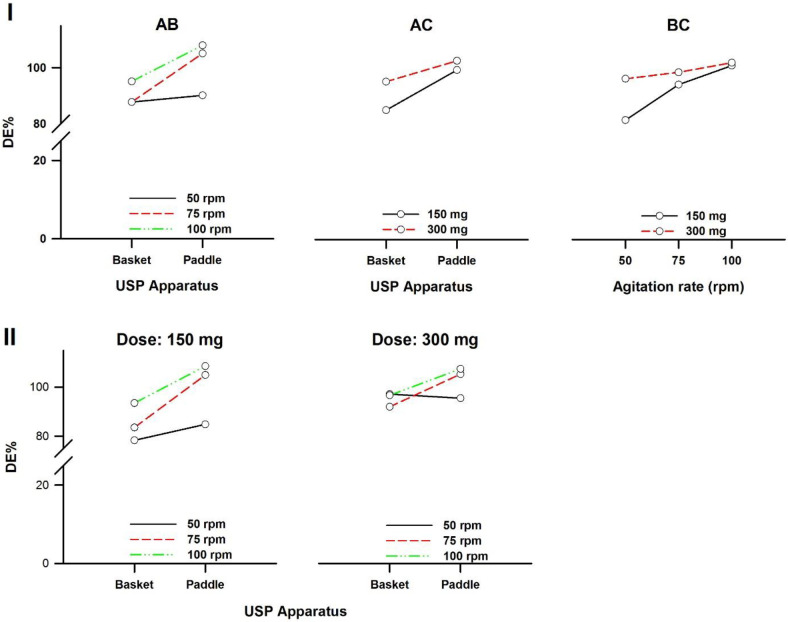
Interaction plots of ranitidine-HCl tablets. (I) First-order and (II) Second-order. A: Dissolution apparatus, B: agitation rate, and C: dose

**Figure 10:**
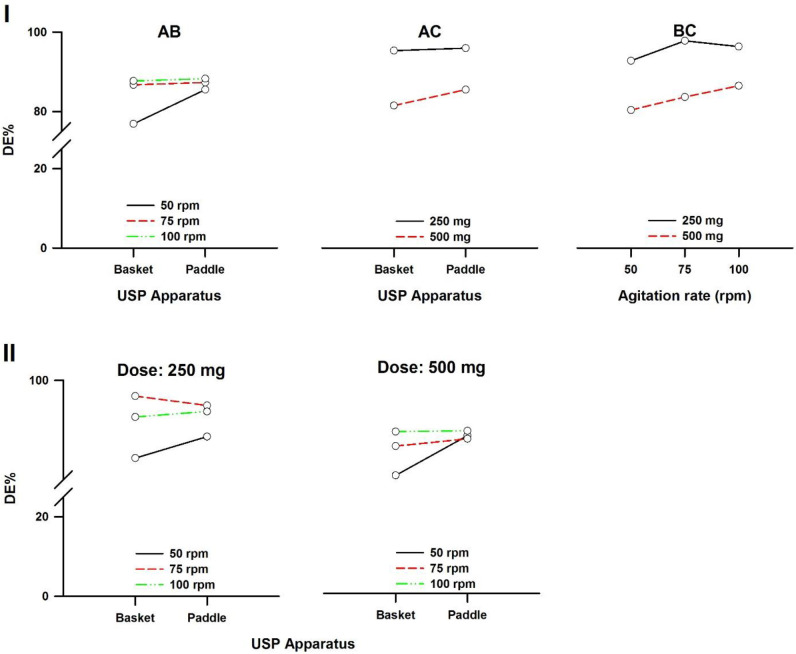
Interaction plots of metronidazole tablets. (I) First-order and (II) Second-order. A: Dissolution apparatus, B: agitation rate, and C: dose

In a three-way ANOVA, there are several effects of interest to be tested. In addition to the main effects of each factor and the interactions found between two of them (called first-order interactions), there may be an interaction between the three factors (a second-order interaction). In other words, there is a first-order interaction when the result of the combination of two factors differs from the sum of the main effects of those factors. There is a second-order interaction when the effect of any first-order interaction is not constant (or not the same) at all levels of the third factor (43). Resulting interaction plots of propranolol-HCl, carbamazepine, ranitidine-HCl, and metronidazole are shown in Figures 7-10, respectively.

The angle of the line joining the two levels in each plot indicates the extent of influence of each dissolution variable on drug release. The plots of the main factors help to identify that the effect of the change in a specific factor has a different influence on drug release among the products (44). This graphical analysis is used to better understand the results of a research involving more than one independent variable (factor) and to avoid making mistakes in the interpretation of the main effects of independent variables. This graphical analysis complements the conceptualization of interaction and how they occurred (45). The hydrodynamic flow characteristics should be considered when using dissolution tests for quality control determinations or for establishing significant *in-vitro/in-vivo* correlations. Under some agitation rate and/or dose conditions, the widely used paddle and basket apparatus produced related hydrodynamic flows around the tablets, resulting in statistically similar parameters. From the point of view of modelling the *in-vivo* environment, *in-vitro* test with hydrodynamic flow characteristics similar to those observed *in-vivo* are required. Since the *in-vivo* hydrodynamic flow is very low (1 mL/min) (46), a dissolution test with a low hydrodynamic flow is preferable. In other words, with the aim of developing adequate dissolution methods to mimic the *in-vivo* performance, the hydrodynamic flow and mechanical stress characteristics of the *in-vitro* performance should be determined.

**Table 1:** Current pharmacopeial conditions of all drug products used

**Drug**	**USP Apparatus**	**Dissolution medium**	**Volume (mL)**	**Agitation (rpm)**	**Wavelength (nm)**	**Q** ** (%)**	**Time (min)**
Propranolol-HCl	1	1% Hydrochloric acid	1000	100	289	75	30
Carbamazepine	2	1% Sodium lauryl sulfate	900	75	285	75	60
Ranitidine-HCl	2	Distilled water	900	50	314	80	45
Metronidazole	1	0.1 N Hydrochloric acid	900	100	278	85	60

**Table 2:** Pharmacopeial tests results, average, n = 10 and model-independent parameters, average ± SEM, n = 6

**Drug**	**Dose (mg)**	**Content uniformity (min-max%)**	**Assay (%)**	**Agitation (rpm)**	**USP**	**Q** ** (%)**	**MDT (min)**	**DE (%)**
A	10	99.7−109.5	102.8	50	1	71.69 ± 0.80	12.16 ± 0.17	42.64 ± 0.72
					2	69.45 ± 1.41	12.94 ± 0.08^*^	39.48 ± 0.75^*^
				75	1	78.38 ± 0.98	13.14 ± 0.12	44.04 ± 0.65
					2	81.94 ± 1.93	13.94 ± 0.14^*^	43.85 ± 0.79
				100	1	89.97 ± 2.23	13.93 ± 0.06	48.19 ± 1.13
					2	90.01 ± 2.72	14.00 ± 0.16	47.97 ± 1.38
	40	110.7−114.7	112.7	50	1	101.56 ± 1.17^†^	12.39 ± 0.29	59.59 ± 0.92^†^
					2	101.12 ± 0.73^†^	11.85 ± 0.34^†^	61.17 ± 1.06^†^
				75	1	101.58 ± 0.95^†^	10.76 ± 0.25^†^	65.13 ± 0.82^†^
					2	100.63 ± 0.72^†^	9.39 ± 0.29^†*^	69.12 ± 0.99^†*^
				100	1	100.57 ± 1.11^†^	6.95 ± 0.32^†^	77.23 ± 0.49^†^
					2	99.53 ± 1.29^†^	8.31 ± 0.41^†*^	71.89 ± 0.92^†*^
B	200	95.5−99.3	98.0	50	1	64.38 ± 2.16	21.60 ± 0.27	41.16 ± 1.14
					2	60.78 ± 1.69	11.75 ± 0.21^*^	48.90 ± 1.49^*^
				75	1	77.32 ± 2.15	17.13 ± 0.46	55.29 ± 1.96
					2	91.55 ± 2.32^*^	9.22 ± 0.33^*^	77.45 ± 1.71^*^
				100	1	62.18 ± 0.53	14.88 ± 0.27	46.76 ± 0.50
					2	102.20 ± 2.09^*^	7.96 ± 0.24^*^	88.61 ± 1.58^*^
	400	93.8−96.1	95.0	50	1	24.67 ± 0.82^†^	25.23 ± 0.68^†^	14.33 ± 0.72^†^
					2	38.50 ± 1.73^†*^	14.39 ± 0.64^†*^	29.36 ± 1.76^†*^
				75	1	44.43 ± 0.44^†^	20.42 ± 0.46^†^	29.31 ± 0.32^†^
					2	90.96 ± 2.01^*^	11.46 ± 1.10^†*^	73.59 ± 1.61^*^
				100	1	52.78 ± 1.69^†^	13.69 ± 0.61	40.69 ± 1.07^†^
					2	101.59 ± 0.75^*^	8.68 ± 0.27^*^	86.90 ± 0.80^*^
C	150	104.3−113.7	108.4	50	1	108.71 ± 0.37	16.78 ± 0.51	78.31 ± 1.11
					2	117.02 ± 1.45^*^	16.52 ± 0.33	84.81 ± 1.45^*^
				75	1	109.67 ± 0.30	14.30 ± 0.09	83.53 ± 0.24
					2	132.93 ± 0.79^*^	12.62 ± 0.19^*^	104.98 ± 0.69^*^
				100	1	117.84 ± 0.33	12.38 ± 0.25	93.53 ± 0.69
					2	132.45 ± 1.12^*^	10.82 ± 0.49^*^	108.58 ± 1.70^*^
	300	91.1−105.5	95.0	50	1	123.00 ± 0.85^†^	12.60 ± 0.37^†^	97.17 ± 1.04^†^
					2	115.18 ± 1.02^*^	10.26 ± 0.45^†*^	95.49 ± 1.25^†^
				75	1	110.60 ± 0.58	10.10 ± 0.38†	91.99 ± 0.88^†^
					2	117.06 ± 0.39^†*^	6.03 ± 0.25^†*^	105.3 ± 0.65^*^
				100	1	110.29 ± 0.58^†^	7.40 ± 0.14†	96.69 ± 0.41^†^
					2	118.09 ± 0.58^†*^	5.40 ± 0.19^†*^	107.46 ± 0.43^*^
D	250	94.9−105.2	95.5	50	1	102.99 ± 0.19	8.58 ± 0.58	88.25 ± 0.94
					2	106.84 ± 0.52^*^	8.61 ± 0.35	91.51 ± 0.37^*^
				75	1	107.36 ± 0.44	5.44 ± 0.27	97.62 ± 0.51
					2	106.23 ± 0.38	5.66 ± 0.19	96.20 ± 0.38^*^
				100	1	101.93 ± 0.41	4.40 ± 0.08	94.46 ± 0.28
					2	102.48 ± 0.37	4.21 ± 0.17	95.29 ± 0.28
	500	96.1−101.0	103.8	50	1	102.84 ± 0.71	21.78 ± 0.60^†^	65.50 ± 1.04^†^
					2	112.17 ± 0.64^†*^	17.47 ± 0.15^†*^	79.51 ± 0.52^†*^
				75	1	107.48 ± 1.11	17.66 ± 0.64^†^	75.81 ± 0.80^†^
					2	104.09 ± 0.73^†*^	14.83 ± 0.85^†*^	78.36 ± 1.49^†^
				100	1	103.86 ± 0.66^†^	13.26 ± 0.27^†^	80.92 ± 0.89^†^
					2	107.61 ± 1.12^†*^	14.75 ± 0.77^†^	81.20 ± 1.98^†^

A: propranolol-HCl; B: carbamazepine; C: ranitidine-HCl; D: metronidazole.

^ †^ *P <* 0.05 (low *vs*. high dose); ^*^P < 0.05 (USP 1 *vs*. USP 2).

Q: Last percentage dissolved of the dissolution test.

**Table 3:** Hyperbola and Weibull parameters. t_63.2%_ and Td values of each fit. Average, n = 6

**Drug**	**Dose (mg)**	**rpm**	**USP**	**Hyperbola parameters**			**t**_63.2%_ ** (min)**	**Weibull parameters**			**Td** ** (min)**
				**a**	**b**	**R** ^2^_adj_		**α**	**β**	**R** ^2^_adj_	
A	10	50	1	138.35	30.09	0.9937	25.24	44.36	1.12	0.9964	25.15
			2	151.65	39.21	0.9729	28.26^*^	1.1_E_^4^	2.29	0.9908	27.18^*^
		75	1	197.34	48.75	0.9838	22.97	1.5_E_^4^	2.39	0.9925	23.08
			2	423.84	124.65	0.9900	22.50	1.2_E_^6^	2.92	0.9949	22.50
		100	1	370.72	96.17	0.9924	19.96	3.1_E_^5^	3.13	0.9891	19.98
			2	2.0_E_^3^	615.16	0.9903	20.15	1.1_E_^7^	3.73	0.9933	20.09
	40	50	1	685.74	154.69	0.9742	15.26^†^	6.7_E_^7^	4.60	0.9954	14.70^†^
			2	361.98	70.41	0.9879	14.49^†^	6.0_E_^6^	3.68	0.9969	14.16†
		75	1	290.71	47.66	0.9636	13.13^†^	1.0_E_^8^	5.16	0.9938	12.96^†^
			2	222.73	25.83	0.9653	10.55^†*^	2.7_E_^6^	4.0	0.9968	11.29^†*^
		100	1	152.92	11.82	0.9248	8.28^†^	1.0_E_^7^	5.07	0.9921	8.34^†^
			2	174.16	17.87	0.9628	9.98^†*^	4.0_E_^6^	3.69	0.9944	10.12^†*^
B	200	50	1	101.83	37.41	0.9930	66.50	31.75	0.83	0.9992	60.78
			2	62.88	5.29	0.9845	268.52	3.53	0.29	0.9956	88.26
		75	1	98.55	18.27	0.9902	33.29	13.17	0.71	0.9995	34.48
			2	99.95	5.36	0.9988	9.33^*^	2.87	0.49	0.9922	9.29^*^
		100	1	67.76	9.36	0.9666	148.26	7.22	0.47	0.9985	66.77
			2	112.42	4.86	0.9942	6.26^*^	5.83	0.83	0.9749	6.40^*^
	400	50	1	55.61	81.11	0.9921	−168.48	133.3	0.84	0.9981	292.06^†^
			2	41.32	8.51	0.9807	−24.34	8.02	0.32	0.9971	611.78^†*^
		75	1	61.67	27.18	0.9988	8.4_E_^3^	22.03	0.62	0.9989	148.57^†^
			2	98.33	6.44	0.9877	11.80	3.23	0.49	0.9980	12.20^†*^
		100	1	57.02	8.13	0.9755	−15.41	6.17	0.37	0.9993	147.84^†^
			2	110.63	4.96	0.9956	6.61	3.31	0.69	0.9938	6.60^*^
C	150	50	1	192.79	36.45	0.9581	17.6	3.1_E_^6^	3.86	0.9677	18.26
			2	171.59	24.27	0.9875	14.16^*^	5.5_E_^3^	2.36	0.9133	14.47^*^
		75	1	168.78	23.90	0.9457	14.30	3.4_E_^6^	4.09	0.9490	15.48
			2	185.68	17.33	0.9618	8.94^*^	1.8_E_^7^	4.45	0.4927	10.70^*^
		100	1	166.86	17.54	0.9291	10.67	3.2_E_^7^	4.62	0.8233	12.52
			2	174.75	13.18	0.9450	7.41^*^	1.2_E_^6^	4.30	0.3897	9.33^*^
	300	50	1	173.57	17.63	0.9425	10.07^†^	2.3_E_^5^	3.72	0.7152	11.74^†^
			2	136.65	8.63	0.9692	7.40^†*^	7.1_E_^3^	2.00	0.8423	8.02^†*^
		75	1	142.94	11.61	0.9618	9.16^†^	9.3_E_^4^	3.41	0.8757	10.60^†^
			2	132.60	4.62	0.9260	4.20^†*^	2.8_E_^3^	3.20	0.5168	5.53^†*^
		100	1	131.32	6.80	0.9061	6.30^†^	5.3_E_^4^	3.78	0.8727	7.81^†^
			2	132.64	4.07	0.9360	3.69^†*^	1.4_E_^4^	3.67	0.3868	5.09^†*^
D	250	50	1	122.82	7.62	0.9187	8.03	47.32	1.58	0.9905	8.25
			2	128.52	7.91	0.9344	7.60	27.46	1.60	0.9600	8.08
		75	1	122.20	4.39	0.8639	4.68	1.6_E_^4^	3.93	0.9079	5.74
			2	121.86	4.71	0.8625	5.06	2.7_E_^6^	5.07	0.9356	6.17
		100	1	113.15	3.25	0.8596	4.11	1.3_E_^6^	5.57	0.9885	4.94
			2	113.13	3.04	0.8657	3.84	2.2_E_^6^	5.46	0.9810	4.75
	500	50	1	267.73	84.42	0.9890	25.76^†^	1.5_E_^5^	2.79	0.9890	25.22^†^
			2	194.56	35.85	0.9750	17.25^†*^	1.5_E_^5^	2.96	0.9526	17.57^†*^
		75	1	202.60	42.45	0.8625	19.03^†^	1.0_E_^6^	3.46	0.9734	19.45^†^
			2	158.65	24.55	0.9678	15.91^†*^	886.01	2.14	0.9893	16.19^†*^
		100	1	150.22	19.54	0.9512	14.17^†^	4.2_E_^3^	2.56	0.9853	14.89^†^
			2	171.21	26.61	0.9388	15.26^†^	3.5_E_^3^	2.60	0.9681	15.59^†^

A: propranolol-HCl; B: carbamazepine; C: ranitidine-HCl; D:metronidazole.

^ †^ *P <* 0.05 (low *vs*. high dose); ^*^P < 0.05 (USP 1 *vs*. USP 2).

**Table 4:** *P*-values for DE data. Significant differences at *P <* 0.05

**Source**	**Propranolol-HCl**	**Carbamazepine**	**Ranitidine-HCl**	**Metronidazole**
A	0.296	< 0.001	0.228	< 0.001
B	< 0.001	< 0.001	< 0.001	< 0.001
C	< 0.001	< 0.001	< 0.001	< 0.001
AB	< 0.05	< 0.001	< 0.001	< 0.001
AC	0.235	< 0.05	0.207	0.059
BC	< 0.001	< 0.001	< 0.001	< 0.001
ABC	< 0.001	< 0.001	< 0.001	0.540

A: Dissolution apparatus; B: agitation rate; C: dose.

## Conclusion

In the present study, four representative BCS drugs manufactured as oral solid dosage forms (reference products) were tested and their dissolution profiles were compared by model-independent, model-dependent, and ANOVA-based comparisons. The three types of comparison revealed significant differences between the two doses of all of the drug products used and between the USP basket and paddle apparatus, excepting for Class I propranolol-HCl at 100 rpm with low dose tablets and 50 rpm with high dose tablets and Class IV metronidazole at 100 rpm with low dose tablets. These results indicate that under these dose and agitation rate conditions, USP Apparatus 1 and 2 produce comparable *in-vitro* results. Conversely, if it is necessary to take advantage of the hydrodynamic differences that each dissolution equipment generates, these conditions should be avoided. Special attention should be paid when biowaiver of low doses of Class I propranolol-HCl and Class III ranitidine-HCl are requested. The information discussed here may be useful for researchers who are involved in formulation development and/or quality evaluations of solid dosage forms. This evaluation should be considered according to the class that each drug represents or by the comparison method used. Variables such as the dose, agitation rate, and type of dissolution apparatus are important factors to consider in designing dissolution tests that reflect the quality of drug products. Further researches on the *in-vitro* release performance of reference drug products are required.
